# Stress vulnerability shapes disruption of motor cortical neuroplasticity

**DOI:** 10.1038/s41398-022-01855-8

**Published:** 2022-03-04

**Authors:** Anne-Kathrin Gellner, Aileen Sitter, Michal Rackiewicz, Marc Sylvester, Alexandra Philipsen, Andreas Zimmer, Valentin Stein

**Affiliations:** 1 Department of Psychiatry and Psychotherapy, University Hospital Bonn, Venusberg-Campus 1, 53127 Bonn, Germany; 2 Institute of Nutritional Science, University of Potsdam, 14558 Nuthetal, Potsdam, Germany; 3 Core Facility Mass Spectrometry, Institute of Biochemistry and Molecular Biology, Medical Faculty, University of Bonn, Bonn, Germany; 4 Institute of Molecular Psychiatry, Medical Faculty, University of Bonn, 53127 Bonn, Germany; 5 Institute of Physiology II, University Bonn, Medical Faculty, Nussallee 11, 53115 Bonn, Germany

**Keywords:** Psychiatric disorders, Learning and memory

## Abstract

Chronic stress is a major cause of neuropsychiatric conditions such as depression. Stress vulnerability varies individually in mice and humans, measured by behavioral changes. In contrast to affective symptoms, motor retardation as a consequence of stress is not well understood. We repeatedly imaged dendritic spines of the motor cortex in Thy1-GFP M mice before and after chronic social defeat stress. Susceptible and resilient phenotypes were discriminated by symptom load and their motor learning abilities were assessed by a gross and fine motor task. Stress phenotypes presented individual short- and long-term changes in the hypothalamic–pituitary–adrenal axis as well as distinct patterns of altered motor learning. Importantly, stress was generally accompanied by a marked reduction of spine density in the motor cortex and spine dynamics depended on the stress phenotype. We found astrogliosis and altered microglia morphology along with increased microglia-neuron interaction in the motor cortex of susceptible mice. In cerebrospinal fluid, proteomic fingerprints link the behavioral changes and structural alterations in the brain to neurodegenerative disorders and dysregulated synaptic homeostasis. Our work emphasizes the importance of synaptic integrity and the risk of neurodegeneration within depression as a threat to brain health.

## Introduction

Chronic stress has been causally linked to neuropsychiatric conditions such as major depressive disorder (MDD) [1, 2]. Deciphering mechanisms of individual susceptibility vs. resilience to stress is only partly understood [3–5], but important to advance the prevention, diagnosis, and treatments of neuropsychiatric disorders. Compared to affective symptoms, motor symptoms and their pathophysiology are understudied in psychiatric patients and their preclinical models. So far, stress research has focused predominantly on limbic and closely connected brain regions, where evidence for disrupted neuronal function underlying affective and cognitive symptoms was demonstrated [6–10]. Meanwhile, motor cortical brain regions and their synaptic properties are insufficiently investigated in stress research.

Glucocorticoids are a pivotal part of the systemic acute and chronic stress response regulated by the hypothalamic–pituitary–adrenal axis (HPAA) and are linked to individual stress vulnerability [11, 12]. This includes a mechanistic role in stress-related changes of behavior and cognition [13]. Moreover, glucocorticoids have been shown to be a relevant influence on synaptic function and stability [14, 15]. The ability to learn and adapt to new tasks requires intact neuroplasticity of the quad-partite synapse composed of the pre- and postsynapse, microglia, and astrocytes [16, 17]. Microglia and astrocytes modulate synaptic strength and stability including postsynaptic spine numbers generally [16] and specifically in the motor cortex [18]. Structural remodeling of glia is an indicator of functional alteration in these cells [19, 20]. Glial alterations in animal stress models and neuropsychiatric patients have been investigated in limbic brain regions and the prefrontal cortex [21–23] but not in detail in the motor cortex.

We here assessed individual vulnerability to chronic social defeat stress by multiple behavioral tests and identified distinct patterns of HPAA response for stress susceptible and resilient mice short- and long-term. By longitudinal in vivo 2-photon imaging we investigated dendritic spine dynamics of layer V principal neurons of the motor cortex in response to stress. With respect to the individual stress phenotype, we identified stress-induced patterns of motor learning (dis)abilities and structural plasticity.

Long-term proteomic changes in the cerebrospinal fluid after CSDS reveal a vulnerability-dependent fingerprint of neurodegenerative disorders and synaptic alterations.

Our study deciphers function and neuroplasticity of the motor cortex with respect to individual vulnerability to chronic stress and highlights it as a new and crucial field at the interface of translational psychiatry and neurology.

## Results

**Fig. 1:**
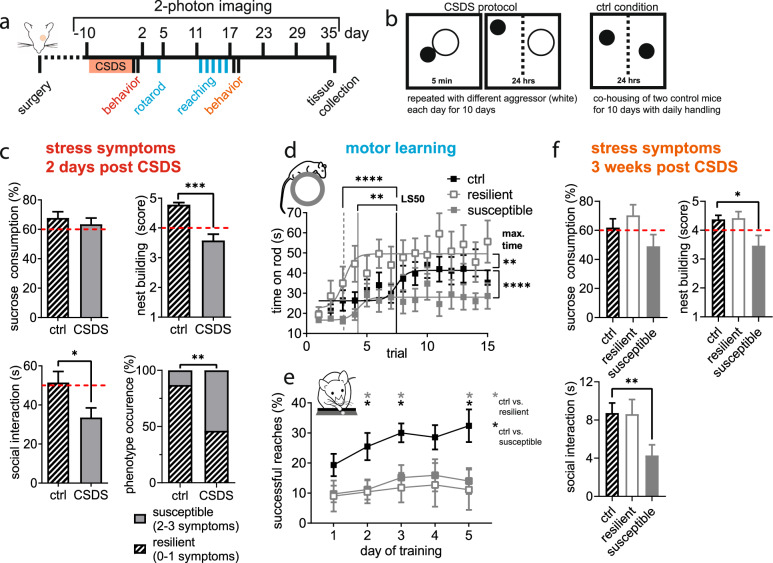
CSDS phenotypes defined by symptom load have distinct alterations in motor learning. **a** Experimental timeline (day 0 defined by the last day of CSDS = first day of behavioral testing). **b** Schematic depiction of the CSDS paradigm and control conditions. **c** Behavioral testing showed reduced nest building (U = 95, *P* < 0.0001, Mann-Whitney U test) and social interaction (t_47_ = 2.399, *P* = 0.021, student’s *t*-test) but no change in sucrose consumption (U = 264, *P* = 0.493, Mann-Whitney U test) in the CSDS group. Individual test results (red dashed lines: cutoff as described in methods) were used for classification as resilient or susceptible phenotype based on symptom load with increased occurrence of the susceptible type after CSDS (*P* = 0.033, Fisher’s exact test); ctrl *n* = 23, CSDS *n* = 26 mice. **d** Susceptible mice failed whereas resilient mice excelled on the accelerating rotarod compared to controls (maximum time: F_2,687_ = 22.03, *P* < 0.0001; learning speed [LS50]: F_2,687_ = 10.08, *P* < 0.0001; one-way ANOVA with Dunett’s post-hoc test). Performance during the first trial did not differ between the three groups (F_2,687_ = 1.574, *P* = 0.208; one-way ANOVA); ctrl *n* = 20, resilient *n* = 12, susceptible *n* = 14 mice. **e** Learning the fine motor task of skilled forelimb reaching over 5 days was impaired in stressed mice (time F_2.209,70.70_ = 3.606, *P* = 0.028; stress F_2,32_ = 5.211, *P* = 0.011; interaction F_8,128_ = 0.762, *P* = 0.637, RM ANOVA with Dunett’s post-hoc test); ctrl *n* = 15, resilient *n* = 7, susceptible *n* = 9 mice (group size reduced by task specific exclusions, see methods for details). **f** Susceptible and resilient stress phenotypes were persistent ~3 weeks after CSDS with only susceptible mice versus controls showing reduced social interaction (H_2_ = 10.22, *P* = 0.006, Kruskal-Wallis test with Dunn’s post-hoc test) and nest building (F_2,43_ = 4.852, *P* = 0.013, one-way ANOVA with Dunett’s post-hoc test). Sucrose consumption did not significantly differ between stressed and control mice as observed before (H_2_ = 3.844, *P* = 0.146, Kruskal-Wallis test); ctrl *n* = 20, resilient *n* = 12, susceptible *n* = 14 mice. *P < 0.05, **P < 0.01, ***P < 0.001, ****P < 0.0001. Results are shown as mean ± SEM.

We stressed adult male mice by the chronic social defeat stress (CSDS) paradigm. After 10 days of CSDS mice were behaviorally characterized regarding stress symptoms. Subsequently, we assessed motor learning in the accelerated rotarod task and the skilled forelimb reaching task (see experimental design in Fig. 1a and b). The sparse GFP-labelling in Thy1-GFP mice allowed for longitudinally studying dynamics of dendritic spines in the primary motor cortex (M1) before and repeatedly after CSDS by 2-photon in vivo microscopy. Over the course of the experiment, we collected plasma, and feces for measuring corticosterone levels. At the end, tissue (brain, adrenal glands) and cerebrospinal fluid (CSF) were collected.

### CSDS-induced susceptible and resilient phenotypes can be defined by symptom load

Stress symptoms were assessed with a behavioral test battery to cover a variety of effects related to chronic stressors: sucrose preference for anhedonia, the nestlet shredding test for self-care, and the social avoidance test (Fig. 1c): CSDS reduced social interaction time and nest building score in stressed mice compared to control animals, whereas sucrose consumption did not differ in the group means; however, individual mice especially in the stressed group showed a clear reduction in sucrose preference (see Supplementary Fig. 1a). This individual variability is not unexpected as the CSDS group consists of the entire vulnerability spectrum (Supplementary Fig. 1a–c). Moreover, different behavioral tests do not necessarily correlate after CSDS in the same animal [24]. In analogy to clinical approaches focusing on multiple symptoms and their severity level, we implemented a combinatory evaluation of symptom load to characterize individual stress vulnerability and to address individual variability in symptom quality and quantity. For each test a cutoff value for pathological results was defined (see methods for details). Exceeding the pathological cutoff in at least two of the three tests after CSDS classified animals as stress susceptible (14/26 mice, 54%) otherwise as stress resilient (12/26 mice, 46%). Mice from control conditions with more than one pathological test result (3/23, 13%) were excluded from further analyses. As expected from the CSDS model, phenotype occurrence differed with a significantly increased frequency of the susceptible phenotype in stressed vs. control animals (Fig. 1c bottom right). Differences in CSDS quality as a cause for the two phenotypes could be ruled out as number and severity of attacks did not differ between resilient and susceptible groups (Supplementary Fig. 2a, b).

With the two stress phenotypes identified we compared motor learning skills between control, resilient, and susceptible mice.

### CSDS-induced phenotypes show distinct patterns of gross and fine motor learning

When gross motor function was challenged on the accelerating rotarod during 15 subsequent trials on day 3 post CSDS (Fig. 1d), learning curves of stressed mice were distinctly different from the controls in terms of maximum time on the rod and learning speed (LS50). Susceptible mice performed very poorly in this task, with a much lower maximum time compared to controls. We noted a shorter LS50 between controls and susceptible mice; however, with respect to the low maximum time of the susceptible group we do not consider this as a relevant learning effect. In stark contrast, resilient mice excelled with a markedly higher maximum time and a significantly faster learning speed compared to controls. Baseline performance indicated by the time on rod in the first trial did not differ between the three groups. In the fine motor learning task, all stressed mice independent of their stress phenotype failed to improve compared to controls (Fig. 1e).

To address symptom load chronification, stress symptoms were re-assessed ~3 weeks after CSDS had ended and after motor learning had been accomplished (Fig. 1f). Phenotype classification did not change within the three groups. Susceptible mice still showed a reduced social interaction time and diminished nest building score. Sucrose consumption remained unchanged compared to controls. We noted a reduction of social interaction time in all groups between the SATs at day one and ~3 weeks post CSDS; however, the relative difference between control and susceptible mice remained. In contrast, the absolute levels of the other tests did not change. Furthermore, the distance travelled by the three phenotypes in the first trial of both SATs, when mice explored the arena without a social stimulus (see methods for details), did not differ (Supplementary Fig. 3a, b) and thus could rule out stress-induced changes in locomotion as a cause for the impaired motor learning.

Next, we sought to confirm stress phenotypes and their chronification by multimodal evaluation of the HPAA as stress effects are linked to a dysregulation of glucocorticoid release [25, 26].

### HPA axis response patterns corroborate stress phenotypes

**Fig. 2:**
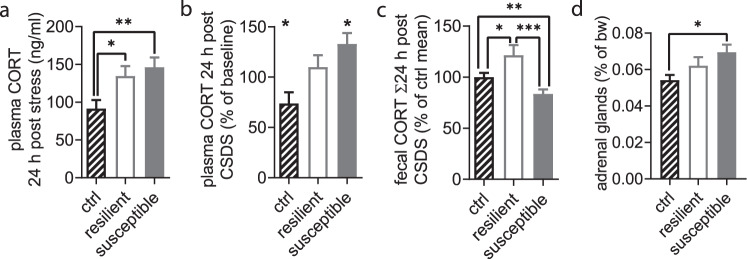
Multimodal HPA axis response after CSDS discriminates stress phenotypes. **a** Plasma corticosterone (CORT) levels were increased 24 h post CSDS (F_2,42_ = 5.954, *P* = 0.005, one-way ANOVA with Holm-Sidak’s post-hoc test); ctrl *n* = 19, resilient *n* = 12, susceptible *n* = 14. **b** Post-stress plasma CORT changed significantly relative to baseline (dashed line) as tested by one sample *t*-tests in controls (t_13_ = 2.365, *P* = 0.034) and susceptible mice (t_8_ = 3.063, *P* = 0.016) but not in resilient ones (t_9_ = 0.843, *P* = 0.421); ctrl *n* = 14, resilient *n* = 10, susceptible *n* = 9. **c** Fecal CORT reflects cumulative release 24 h after CSDS and was significantly different between stressed phenotypes and stressed vs. control mice (F_2,41_ = 7.897, *P* = 0.001, one-way ANOVA with Holm-Sidak’s post-hoc test); ctrl *n* = 20, resilient *n* = 12, susceptible *n* = 12 mice. **d** Adrenal gland weight measured 36 days post CSDS was significantly higher in the susceptible mice compared to controls and resilient mice (F_2,41_ = 4.580, *P* = 0.016, one-way ANOVA with Holm-Sidak’s post-hoc test); ctrl *n* = 19, resilient *n* = 12, susceptible *n* = 14 mice. *P < 0.05, **P < 0.01, ***P < 0.001. Results are shown as mean ± SEM.

Corticosterone in plasma sampled 24 h after the last social defeat session differed significantly between the groups (Fig. 2a). Hormone levels were significantly elevated in both resilient and susceptible mice. Baseline corticosterone sampled before the stress phase did not differ between the groups (Supplementary Fig. 4a). When we compared post-stress corticosterone levels with the individual’s prestress value (Fig. 2a), susceptible mice presented a significant increase from baseline, but not resilient individuals (Fig. 2b). Moreover, control mice showed a significant decrease of plasma corticosterone in the pre/post comparison.

Physiological diurnal corticosterone release is necessary for motor cortical neuroplasticity [27]. Hence, we also investigated cumulative fecal corticosterone levels from the 24 h following the last defeat. We found significant differences between stressed and unstressed mice (Fig. 2c,) with lower 24 h fecal corticosterone release in susceptible and higher levels in resilient mice. This resulted in a marked difference between the two stress phenotypes. The stress chronification in the behavioral domain was further corroborated by an increased absolute and relative weight of the adrenal glands 36 days post-stress (Fig. 2d and Supplementary Fig. 4b) in susceptible, but not resilient animals. These data demonstrate that the system wide HPAA response was clearly dependent on individual stress vulnerability and persisted for more than five weeks after the stress period had ended.

HPAA dysregulation has been shown to affect motor learning and motor cortical spine dynamics [27–29]. In the next step, we assessed spine dynamics of GFP-labelled layer V principal neurons by longitudinal in vivo imaging.

### CSDS disrupts motor cortical spine dynamics with phenotype-dependent recovery

**Fig. 3:**
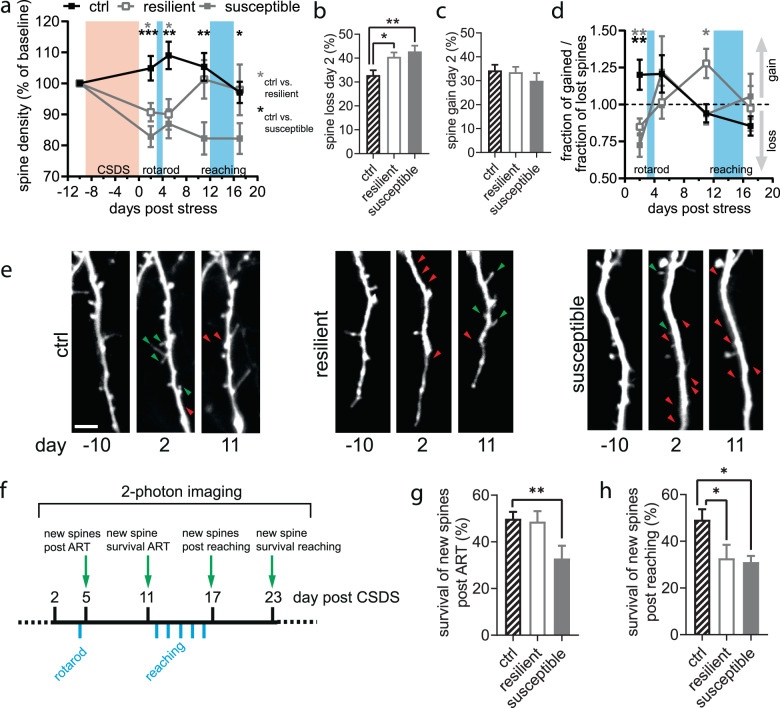
Response and recovery of motor cortical spine dynamics after CSDS. **a** Development of spine density from day 2 until 17 post CSDS (time F_3.155,163.3_ = 3.425, *P* = 0.017, stress F_2,64_ = 8.324, *P* = 0.0006, Interaction F_8,207_ = 4.211, *P* = 0.0001, RM ANOVA mixed model with Dunett’s post-hoc test). **b**, **c** Change in spine loss but not spine gain drives the change in spine density on day 2 (loss: F_2,64_ = 6.784, *P* = 0.002, one-way ANOVA with Dunett’s post-hoc test, gain: F_2,64_ = 0.767, *P* = 0.487, one-way ANOVA). **d** Net gain or loss between imaging sessions is differently influenced by stress over time (time F_2.303,109.8_ = 2.221, *P* ≥ 0.05, stress F_2,51_ = 0.347, *P* ≥ 0.05, interaction F_6,143_ = 3.164, *P* ≤ 0.01, RM ANOVA mixed model). **e** Example time lapse images of dendritic spines at baseline (day −10), day 2 and 11 post CSDS (green arrowhead = new spine, red arrowhead = lost spine). Scale bar: 5 µm). **f**, **g** Survival fraction of spines formed after the different motor learning tasks (rotarod [ART]: F_2,48_ = 4.918, *P* = 0.011, reaching: F_2,31_ = 4.477, *P* = 0.020, both one-way ANOVA with Dunett’s post-hoc test). **a**–**d**, **g**, **h**: no. of ROIs/mice from day −10 to 17: ctrl 26/19 to 23/12, resilient 22/10 to 10/5, susceptible 16/10 to 13/8, see also Supplementary Table 1). *P < 0.05, **P < 0.01, ***P < 0.001. Results are shown as mean ± SEM.

We imaged dendritic spines of layer V neurons of the primary motor cortex one day before and 2, 5, 11, and 17 days after CSDS. Spine densities were profoundly altered by stress over time in both resilient and susceptible mice when compared to the control group (Fig. 3a, e). At 2 days post CSDS resilient and susceptible mice showed a significant drop in spine density compared to control (ctrl: 104.91 ± 3.99%, resilient: 90.74 ± 2.95%, susceptible: 82.87 ± 3.37%). This effect was predominantly driven by a loss of spines (Fig. 3b), whereas spine gain was unaffected (Fig. 3c). Eleven days post CSDS spine density had recovered to control levels in resilient, but not in susceptible mice (Fig. 3a, e).

Isolated evaluation of spine gain and loss over time (Supplementary Fig. 5a-b) did not sufficiently decipher the alterations in the gain/loss balance that led to the changes in spine density of each stress phenotype. Consequently, we compared the gain/loss ratio (GLR, fraction of gained/fraction of lost spines, <1 = net loss, >1 = net gain) and its dynamic over the time course of the experiment (Fig. 3d) and found a significant interaction of stress and time. The recovery of spine density in resilient mice by day 11 was driven by a significantly elevated gain/loss ratio (Fig. 3d). In a subset of mice, we re-imaged dendritic spines at day 23, 29, and 35 after CSDS. While resilient animals had returned to control levels as early as day 11 post-stress, susceptible mice reached this level only at day 23 (Supplementary Fig. 5c).

### CSDS alters spine stability underlying successful motor learning

Changes in motor cortical spine dynamics have been observed during and after gross and fine motor skill training [30, 31] with the requirement of stabilization of newly formed spines. Therefore, we analyzed the stability of those spines that had been newly formed after each motor task (Fig. 3f). Whereas resilient mice showed the same stability rate as controls after the accelerated rotarod training (ART), the susceptible group which had failed to learn the task showed a significantly decreased survival of these newly formed spines (Fig. 3g). Similarly, when looking at the stability of spines formed during the skilled forelimb reaching, we found that the groups without learning success demonstrated decreased survival of new spines compared to the successfully trained control group (Fig. 3h).

### CSDS induces long-term glial changes linked to altered neuroplasticity and neuroinflammation

**Fig. 4:**
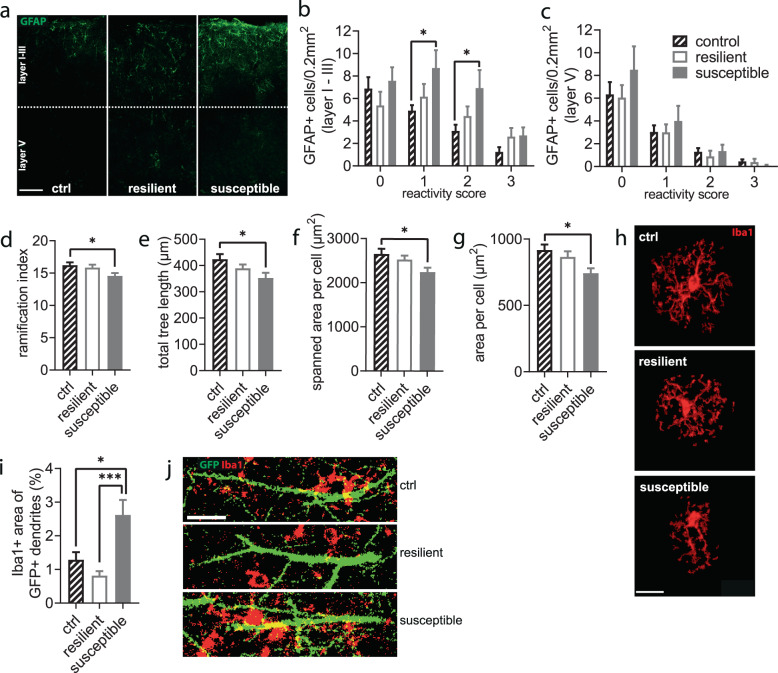
Motor cortical glia cells show long-term response to CSDS. **a**–**c** Layer- and phenotype-specific reactivity of astrocytes (Layer I–III: stress F_2,220_ = 6.500, *P* = 0.002, reactivity score F_3,220_ = 14.30, *P* < 0.0001, interaction F_6,220_ = 1.140, *P* = 0.340, layer V: stress F_2,220_ = 0.999, *P* = 0.370, reactivity score F_3,220_ = 36.78, *P* < 0.0001, interaction F_6,220_ = 0.509, *P* = 0.801, two-way ANOVA with Holm-Sidak’s post-hoc test when applicable). No. of ROIs/mice: ctrl *n* = 26/13, resilient *n* = 18/9, susceptible *n* = 14/7. **d**–**h** Morphological changes of Iba1+ microglia cells in layer I–III are limited to the susceptible group (ramification index: F_2,121_ = 3.148, *P* = 0.047, tree length F_2,121_ = 3.576, *P* = 0.031, spanned area F_2,121_ = 3.459, *P* = 0.035, total area F_2,121_ = 4.061, *P* = 0.020, one-way ANOVA with Holm-Sidak’s post-hoc test). No. of cells/mice: ctrl *n* = 48/11, resilient *n* = 45/9, susceptible *n* = 31/6. **i**, **j** Microglia-dendrite colocalization was significantly increased in susceptible mice compared to controls but also markedly increased compared to resilient mice (H_2_ = 14.69, *P* = 0.0006, Kruskal-Wallis test with Dunn’s post-hoc test). No. of dendrites/mice: ctrl *n* = 43/11, resilient *n* = 47/9, susceptible *n* = 36/6). Scale bar: 200 µm in a, 20 µm in **h**, **j**. *P < 0.05, ***P < 0.001. Results are shown as mean ± SEM.

Astrocytes and microglia form a functional unit with dendritic spines in the quad-partite synapse and can modulate synaptic strength and stability [16]. The dramatic effects on spine dynamics together with the chronic changes in behavior and HPAA prompted us to study astrocyte and microglia morphology in the brains harvested 36 days post CSDS. First, we analyzed the deeper motor cortical layer V and superficial layers I–III separately regarding their immunoreactivity for the astrocytic glial fibrillary acidic protein (GFAP), as the deeper layer contains the somata of the principal neurons that extend their dendrites to the superficial layers. Analysis of the superficial layers revealed astrogliosis in susceptible mice (Fig. 4a) indicated by higher numbers of GFAP-positive cells (Supplementary Fig. 6a) accompanied by an increased reactivity measured by a morphological score (see methods and Supplementary Fig. 6d for details) (Fig. 4b). In contrast, deeper layer V remained unaffected by the stress exposure regarding cell numbers (Supplementary Fig. 6b) and reactivity score (Fig. 4c).

Second, staining for the microglial marker ionized calcium binding adaptor molecule 1 (Iba1) revealed several morphological alterations of microglia in the superficial layers I–III of susceptible, but not resilient mice (Fig. 4h). We found ramification index (Fig. 4d) and tree length (Fig. 4e) as well as spanned area (Fig. 4f) and total area (Fig. 4g) per cell decreased. Guided by these chronic changes in microglia we asked whether those changes would also point at an altered interplay between microglia and neuronal structures, i.e., dendrites and spines in layer I–III of the motor cortex, which we had imaged during the in vivo experiments. We found a significantly increased colocalization of GFP positive dendrites and Iba1 positive microglia in susceptible mice but not in resilient mice compared to controls (Fig. 4i-j). Strikingly, in resilient and susceptible mice the colocalization of microglia and dendrites differed even more prominently. Iba1 coverage in the analyzed cortical regions did not differ between groups (Supplementary Fig. 6c).

These results again indicate chronic changes in regulatory pathways of neuronal and non-neuronal cells of the brain. To address this question further, we employed total proteomic analysis of the cerebrospinal fluid (CSF) of the mice as CSF is also a frequently assessed sample in neuropsychiatric disorders.

### CSDS leaves long-term proteomic fingerprints linked to neurodegeneration and synapses in CSF

**Fig. 5:**
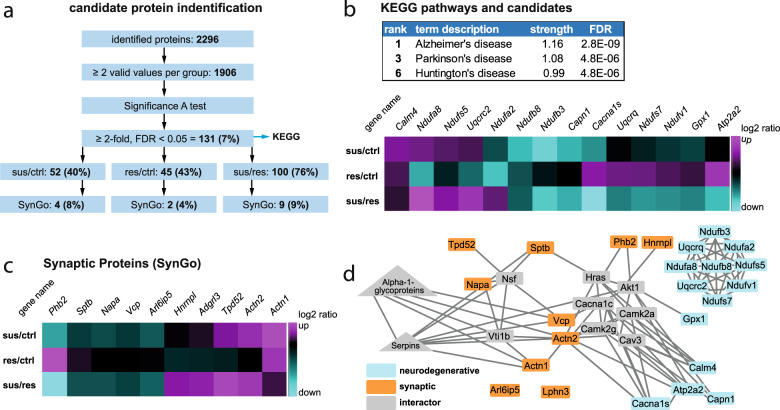
Proteins linked to neurodegeneration and synapses are regulated long-term in CSF after CSDS. **a** Workflow for identification of regulated proteins from the CSF sample set (ctrl *n* = 17, resilient *n* = 11, susceptible *n* = 10 mice). **b** Significant KEGG pathway results for neurodegenerative disorders and their regulated protein groups (gene names shown). **c** Regulated synaptic protein groups by SynGo database annotation (gene names shown). **d** Interaction network of the regulated candidates from the neurodegenerative and synaptic annotations.

CSF was drawn immediately before brain harvest 36 days after CSDS. Mass-spectrometry of individual samples and analysis using a data-independent acquisition approach (DIA) identified 2296 different protein groups and 1906 protein groups after filtering for ≥2 valid values per group. A total of 131 regulated protein groups (≥2-fold change, FDR < 0.05) emerged from the three intergroup comparisons (sus/ctrl, res/ctrl, sus/res). The majority of the regulated protein groups (76%) differed between samples from susceptible and resilient animals. 40% and 34% differed between susceptible versus control and resilient versus control samples, respectively (Fig. 5a). Interestingly, the regulated protein groups showed KEGG (Kyoto Encyclopedia of Genes and Genomes) pathway enrichment for the three neurodegenerative disorders Alzheimer’s, Parkinson’s, and Huntington’s disease. In total, 14 protein groups in the dataset belonged to these KEGG pathways and protein number and direction of regulation differed between the three comparisons. This resulted in distinct patterns for stress phenotypes versus controls but also between the phenotypes (Fig. 5b). Half of these protein groups are part of the mitochondrial oxidative phosphorylation system (OXPHOS), as subunits of complex I (NADH dehydrogenase, Ubiquinone, *Nduf*). There was no overlap between all regulated protein groups in sus/ctrl comparison (*Capn1, Calm4, Ndufb3, Ndufb8*) and res/ctrl comparison (*Atp2a2, Cacna1s, Ndufa2, Ndufa8, Ndufs7, Uqcrq*). For sus/ctrl, all of the four regulated protein groups except *Calm4* were strongly downregulated (>4-fold). For res/ctrl, only *Ndufa2 and* eight showed downregulation whereas the remaining four protein groups where upregulated. Most candidates emerged from the comparison between susceptible and resilient samples (*Atp2a2, Cacna1s, Capn1, Gpx1, Ndufa8, Ndufb3, Ndufs5, Ndufs7, Ndufv1, Uqcrc2, Uqcrq*) with upregulation of the mitochondrial *Ndufa8, Ndufs5,* and *Uqcrc2* and downregulation seen in the remaining eight protein groups.

Since we focused on stress affecting structural neuroplasticity underlying behavioral consequences, we next looked for regulation of synaptic proteins detected by annotation via the SynGo database [32]. A set of 10 protein groups was identified with distinct patterns of up and downregulation for each of the comparisons (Fig. 5c). The susceptible group (vs. ctrl) showed upregulation for Alpha-actinin-1 (*Actn1*) and -2 (*Actn2)* and downregulation for the PRA1 family protein 3 (*Arl6ip5*) as well as Prohibitin-2 (*Phb2*). In contrast, the resilient group (vs. ctrl) had a marked upregulation for Prohibitin-2, whereas Alpha-actinin-1 was upregulated as also found in susceptible samples vs. control. Comparison between the stress phenotypes revealed nine protein groups to be up or downregulated. The majority of these synaptic components was relatively downregulated in susceptible vs. resilient individuals: Alpha-soluble NSF attachment protein (*Napa*), Spectrin beta chain (*Sptb*), Transitional endoplasmic reticulum ATPase (*Vcp*) and as well as Prohibitin-2 (*Phb2)* and PRA1 family protein 3 (*Arl6ip5)*. Four candidates, Tumor protein D52 (*Tpd52*), Alpha-actinin-2 (*Actn2*), Heterogeneous nuclear ribonucleoprotein L (*Hnrnpl*) and Adhesion G protein-coupled receptor L3 (*Adgrl3*) were upregulated in the res/sus comparison. Network analysis [String database version 11[33]] revealed links between the neurodegenerative and synaptic candidates (Fig. 5d) via interactors for regulating calcium homoestasis (*Camk2a, Camk2g*), integral membrane proteins involved in receptor endocytosis (*Cav3)* and the RAC-alpha serine/threonine-protein kinase (*Akt1*).

## Discussion

We studied long-lasting detrimental consequences of 10 days of chronic social defeat stress on motor cortical neuroplasticity. We used a combination of behavioral tests to classify stressed animals as stress susceptible and stress resilient. We used motor learning paradigms to asses functional changes of the motor cortex. We demonstrate distinct patterns of HPAA (dys)function. To decipher underlying mechanisms of altered learning in stress susceptible, resilient and control mice we analyzed spine dynamics by in vivo imaging. Histology of the motor cortex revealed long-term changes of microglia cells and astrocytes.

We discovered significant differences between stress-susceptible and stress-resilient groups in all modalities studied.

### Multimodal behavioral classification of stress vulnerability aids demasking of complex stress effects

Chronic social defeat stress (CSDS) is a valid animal model of depression that has been proven to inflict severe and lasting symptoms mainly tested in the social behavior domain [34, 35]. In general, a subset (~30–40%) of the mice subjected to CSDS show no or mild behavioral change, mostly investigated with the social avoidance test. These mice, although termed resilient, can also exhibit other behavioral and endocrinological stress symptoms [3]. Consequently, relying on a combination of behavioral tests results in a more robust classification [36] and translates better to the clinical practice in humans, where several behavioral symptoms are evaluated for a diagnosis of a psychiatric condition like major depression or anxiety disorders [37]. With this in mind, we developed a cutoff based categorial approach with three behavioral tests for anhedonia, self-care, and social interaction to discriminate two subpopulations of mice based on symptom load after stress. These subpopulations showed distinct, individual patterns in all subsequent experiments. Symptom persistence is another important criterion for diagnosing chronic mental disorders in humans and was demonstrated in CSDS before [3]. In line, after ~3 weeks, symptom load was also confirmed in our stress phenotypes and links stress-induced behavior, long-term systemic and central-nervous cellular and molecular changes seen as late as 5 weeks post-stress in our dataset.

Stress activates the HPA axis. Our results confirm previous studies, which also showed an elevation of plasma corticosterone levels in both susceptible and resilient stress phenotypes [3]. Interestingly, when normalizing values to the value before stress only susceptible mice had a significant elevation of plasma corticosterone, suggesting a relevant threshold for individual disturbance of homeostatic HPAA balance.

Stress-induced hyperactivation of the HPAA including increased weight of adrenal glands as the source of corticosterone was demonstrated in several social stress models [3, 38] as well as in patients suffering from MDD [39]. In accordance, we found increased acute plasma levels. The reduced cumulative fecal corticosterone 24 h post CSDS and increased adrenal weight 5 weeks after CSDS depict a long-lasting pathological and insufficient HPAA function in susceptible mice which exhibited the most critical set of behavioral, motor functional, and neuroplastic impairments. Both in animal models and humans, such as PTSD patients, chronic stress has been linked to insufficient glucocorticoid signalling and diminished diurnal corticosterone release [40–44].

In addition, we revealed lowered plasma corticosterone in control mice after the stress period. Together with the slight increase of spine density (~5%) in controls this could indicate an effect on basal motor behavior in the new housing condition, effects of sensory co-housing with a non-aggressive littermate, and/or the effect of daily handling. It also emphasizes the relevance of matching control conditions to avoid over- or in our case potential underestimation of biological readouts in behavioral stress models.

### Spine loss and recovery after CSDS are linked to HPA axis stress response

By longitudinal in vivo imaging we observed a severe drop of dendritic spine density in the primary motor cortex after CSDS regardless of the individual symptom load. The here reported reduction in spine density (−9% in resilient to −17% in susceptible from their baseline, −14% and −22% compared to control levels) is relatively high if one compares it to changes we observed by direct manipulation of synaptic adhesion [45]. Detailed analysis of spine dynamics revealed that changes in spine density were driven by spine loss. Synaptic loss is a common neuropathological finding in psychiatric disorders like MDD [46] but also in the neurodegenerative Alzheimer’s disease [47, 48]. In behavioral preclinical stress models, synapse loss has been reported before in other cortical brain regions such as prefrontal cortex in a range of 4–16% [6, 7]. The Gan group also focusing on the motor cortex utilized systemic corticosterone treatment to mimic stress and also saw spine loss and an impaired ability to learn a gross motor task on the rotarod [27]. Moreover, physiological corticosterone levels are a general prerequisite for successful stress coping and learning [27, 49]. To date, spine dynamics of the motor cortex after CSDS were only described in a small control group by Shu et al. [7]. They focused on the frontal association cortex and found only a small reduction (4% from control levels) driven by impaired spine formation but not enhanced spine loss. Opposed to our results, they reported no stress-induced change in spine density or dynamics in the motor cortex [7]. However, results are hard to compare, as they did not separate susceptible and resilient animals and imaged less frequently.

### Structural neuroplasticity altered by stress is linked to impaired motor learning

Besides altered spine dynamics, glucocorticoids as a mimic of stress have been shown to affect both motor performance and gross motor learning [27, 29]. By combining gross and fine motor task learning subsequently in each mouse, we accomplished an intraindividual characterization of motor learning alterations with respect to individual stress vulnerability. Our results show conclusively that stress-susceptible individuals lose the ability to learn both a gross and fine motor skill, whereas gross motor learning is improved in resilient individuals but cannot compensate for a loss of fine motor skill learning abilities. Especially the forelimb reaching task in mice mimics to a limited extent the refined use of fingers and the hand, a complexity mostly unique to humans and primates [50, 51]. Hence, by revealing the detrimental effects of chronic stress to this crucial human asset our results could guide future clinical diagnosis and treatment focus neglected to date.

Changes in spine density in M1 are a prerequisite of fine and gross motor skill learning as demonstrated previously in mice not challenged by stress [30, 31]. Successful learning requires persistence of newly formed spines after training [30, 31, 52, 53]. In line with this, the two phenotypes of stressed mice in our study had also only shown learning success when spine persistence occurred afterwards. Susceptible mice presented task-independent severe disruption of neuroplasticity whereas resilient mice showed task-dependent learning capability after stress. It is tempting to speculate, that rotarod learning abilities after stress could be part of predisposing traits of stress vulnerability rather than a sole consequence. Stress-induced lack of motivation as a cause for the differences in learning success are unlikely due to the design of the habituation and training phases in the motor tasks (see method section).

### Chronic glial changes in the primary motor cortex point to a disturbed quad-partite synapse

Since behavioral and endocrine stress effects persisted long after spine density levels had recovered, one could speculate that the restoration of synapse number might not be a bona fide sign of recovery after stress.

Chronic glial changes in the motor cortex after stress are not well characterized. In few studies, M1 served as a negative control region for the PFC [54, 55]. In studies investigating the PFC, where spine loss was also observed after chronic stress as discussed before, both astrocytic loss but also morphological remodeling with e.g., reduction in branch number had been detected [54]. Varying CSDS protocols and observation periods are likely underlying differences in reported glia response in stress as suggested elsewhere [23] and thus limiting comparability with our data. Also, other studies did not differentiate between different stress phenotypes, which could mask the findings we were able to detect in the motor cortex by single-cell evaluation in different cortical layers in the stress subgroups. It has been hypothesized that astrocytic loss/atrophy could be a consequence of glutamatergic hyperactivity in the PFC [15, 54] and in turn contributes to neuronal damage by reducing glutamate clearance [56]. Our data of unchanged (resilient) or increased (susceptible) cell numbers indicates that astrocytes in M1 could still be affected by but also compensating neuronal glutamate excess, supporting neuronal protection, and recovery. An electrophysiological ex vivo study showed layer-dependent change in neuronal activity in rat M1 after 10 days of high doses of corticosterone administration in vivo, with enhanced excitatory input in layer II/III but not layer V [57]. Although their pharmacological stress model cannot serve as a direct comparison to our behavioral model also given the phenotype-specific alterations of the HPA axis we detected, it can be speculated that layer-dependent enhanced glutamatergic transmission contributes to the morphological changes in astrocytes observed by us.

Both psychiatric and neurodegenerative disorders have glial and especially microglial impairments contributing to their development [22, 58]. The present study found morphological signs of microglia activation ~5 weeks post CSDS, similar to those found together with neuroinflammatory markers in limbic brain regions post CSDS [23], that can contribute to spine loss [59] as also seen in neurodegenerative diseases [58]. Microglia interact with dendritic spines in vivo [60] and can lead to stabilization or pruning of synapses [59, 61] and are therefore crucial for general but also specifically motor cortical plasticity [18]. In this light, our data of increased microglia-dendrite colocalization and reactive microglia morphology exclusively in susceptible mice indicates neuroinflammatory processes involved in the structural and functional impairment of the motor cortex after chronic stress.

### Neurodegenerative and synaptic fingerprints in CSF after CSDS

CSF analysis in animal models of stress is considerably rare despite the translational value. It is a frequent diagnostic tool in humans presenting with neuropsychiatric symptoms and disorders and gives direct access to neurodegenerative, inflammatory, and neuronal markers [62] that often cannot be detected in other specimens like plasma due to the blood–brain barrier. We detected enriched pathways from three neurodegenerative disorders (Alzheimer’s, Parkinson’s and Huntington’s disease) in chronically stressed mice using the KEGG database, which is in line with cumulating evidence for links between neurodegenerative disorders and depression in humans [63]. A subset of the regulated proteins we found annotated for neurodegenerative disorders after stress belonged to the mitochondrial OXPHOS system. Mitochondrial dysfunction has been detected not only in both psychiatric [64] but also neurodegenerative disorders [65], underlining the links between the two entities. The mitochondrial complex 3 subunit *Uqcrc2* upregulated in susceptible versus resilient mice has been implicated in potentially protective/restorative strategies to oxidative and neuroinflammatory stress [66].

We also found a distinct subset of synaptic protein groups regulated in CSF after CSDS. The most prominent difference was seen in Prohibitin-2 (*Phb2*) levels with downregulation in susceptible and upregulation in resilient mice. It had previously been shown to be reduced in the hippocampus of rats after a single social defeat [67] and might thus be a sensitive marker for this type of stress. Prohibitin-2 is a receptor crucial for Parkin-induced mitophagy [68], regulates inflammation and a neuron-specific deletion had resulted in neurodegeneration [69, 70]. This might suggest higher mitophagy capacity in resilient mice compensating neuroinflammation and neurodegeneration. Alpha-actinin 1 and 2 have been linked to integrity of synapses as part of the postsynaptic density [71], regulating spine numbers, size, and remodeling [72, 73], and the pronounced upregulation of alpha-actinins in susceptible mice matches well with their protracted recovery of spine numbers. PRA1 family protein 3 (*Arl6ip5*), a modulator of glutamate transporters in synapses [74, 75] is also found in astrocytes [76]. A neuroprotective, anti-inflammatory role was demonstrated for astrocytic PRA1 family protein 3 in a Parkinson’s disease model [76]. The downregulation of *Arl6ip* in susceptible mice (vs. control and resilient mice) who had also shown increase in GFAP + cells in line with the data from Miao and colleagues [76] might again point to impaired anti-inflammatory capacity in this phenotype.

Stress response is a spectrum of changes that presumably hold individual thresholds for a negative impact on the organisms. Hence, relative changes between the two behavioral stress phenotypes might point to a disbalance of synaptic homeostasis. A set of synaptic proteins was exclusively regulated in the comparison between susceptible and resilient mice. Both spectrin beta (*Sptnb)* and the transitional endoplasmic reticulum ATPase (*Vcp*) are relevant for the control of structural synaptic plasticity [77, 78] and more specifically dendritic spine formation [79], and were downregulated in susceptible versus resilient mice. *Napa*, the alpha-soluble NSF attachment protein, is a direct interactor with SNAP-25 in the SNARE complex at the presynapse; SNAP-25 downregulation is linked to impaired synaptic plasticity and spine morphogenesis [80]. *Adgrl3* (Laterophilin-3), a neuronal adhesion-GPCR, regulates synaptic density and layer 2/3 synaptic input to layer 5 [81] and was relatively reduced in resilient vs. susceptible samples. Whether this points to a counteracting mechanism in resilient mice in the light of a potential glutamatergic hyperactivation of layer 2/3 [57] in M1 remains to be elucidated. To further support the theory of glutamatergic disbalance in the cortex, tumor protein D52 (*Tpd52*) was significantly differing between the susceptible (up) and resilient group (down) vs. controls. This protein has been identified as part of glutamatergic synaptosomes in the pre- and postsynapse [82] and its upregulation in susceptible mice could be indicative of the deregulated, hyperactive glutamatergic signaling suggested in the pathogenesis of MDD discussed before but also AD [83].

## Conclusion

Our findings are in line with the understanding of CSDS as a robust model of affective neuropsychiatric disorders and inductor of neurodegeneration and glial activation. Here we demonstrate that chronic social stress affects strongly the motor cortex and its function.

We refined the characterization of resilient and susceptible mice in the CSDS model by evaluating three different behavioral domains. We found detrimental impairment of fine motor learning and motor cortical spine loss in stress resilient, phenotypically healthy mice. With this in mind, stress sequelae presenting as motor deficits in humans should be included in the diagnostic and therapeutic pathways as well as critical reevaluation of the meaning of resilience after stressful, traumatic periods in life. Together with our proteomic data the cellular changes seen in neurons and glia cells might lead to new approaches towards deciphering neuropsychiatric conditions such as MDD as potentially neurodegenerative disorders with persisting changes in synaptic structure and function.

## Material and methods

### Animals

Fifty four adult male mice (age 10 ± 0.75 weeks at surgery) with sparse expression of enhanced green fluorescent protein (EGFP) in pyramidal neurons in layer 5 and more rarely layer 2/3 (Thy1-GFP M [84], RRID: IMSR_JAX:007788) were used in a total of 5 cohorts (replications of the whole experiment). Mice were group housed (2–5/cage) prior to surgery and single housed throughout the entire experiment except for the chronic stress period. Mice were fed ad libitum and maintained in the same room under a 12:12 h light/dark cycle at constant temperature (22 °C). All experiments followed the guidelines of the German Animal Protection Law and have been approved by the government of North Rhine Westphalia (Local Committee for Animal Health, LANUV NRW).

### Cranial window surgery

Mice were deeply anaesthetized (medetomidin 0.5 mg/kg, midazolam 5 mg/kg, fentanyl 0.05 mg/kg bodyweight i.p.) and received carprofen 5 mg/kg s.c. for perioperative analgesia. A craniotomy 3–4 mm in diameter was carefully drilled over the right or left primary motor cortex, depending on the paw preference determined prior to the surgery. A round glass coverslip 5 mm in diameter was placed and sealed with cyanoacrylate and dental acrylic over the craniotomy (Fig. 2a). A custom plastic bar was attached to the parietal bone contralateral to the trepanation allowing for fixation of the head during microscopy. Anesthesia was antagonized (atipamezol 2.5 mg/kg, flumazenil 0.5 mg/kg, naloxone 1.2 mg/kg bodyweight i.p.) and mice allowed to return to full alertness and normal mobility under a warming lamp. Buprenorphine 0.1 mg/kg bodyweight was applied 2×/d s.c. for 3 days starting 12 h post-surgery for analgesia.

### 2-photon in vivo imaging

3.29 ± 0.13 weeks post-surgery mice were slightly anaesthetized (medetomidin 0.5 mg/kg, midazolam 5 mg/kg bodyweight i.p.) to ensure immobilization during the imaging session. Body temperature was kept constant at 37 °C by a heated microscope stage. The head was fixed in a custom-made holder and the cranial window was aligned horizontally. A custom build 2-photon microscope equipped with a Chameleon Vision S laser (Coherent) and a water immersion objective lens (40x, NA 0.8, Olympus) was used for in vivo imaging. Images were acquired using ScanImage software (Vidrio Technologies) [85]. Excitation wavelength was tuned to 910 nm for imaging of GFP. Image stacks (xy-dimension: 512 × 512 px) of one to three regions of the motor cortex containing several GFP positive dendritic segments were imaged at a resolution of ~0.08 µm/pixel (x, y), z-step size of 0.8 µm and a pixel dwell time of 2000 ns. The regions were re-imaged up to seven times over the course of the experiments with a minimum interval of 3 and a maximum interval of 12 days (see Fig. 1a). After imaging, anesthesia was antagonized with atipamezol 2.5 mg/kg and flumazenil 0.5 mg/kg bodyweight i.p and mice were allowed to fully recover under a warming lamp.

### Chronic social defeat stress (CSDS)

Mice were randomly assigned to either stress or control treatment with a ratio of 1.5:1 for treatment group size. Mice in the stress group were subjected to 10 consecutive days of chronic social defeat stress as previously described [34]. Each day, the experimental mouse (intruder) was introduced to the home cage of another aggressive, bigger CD1 mouse (resident) and exposed for 5 min to physical attacks and threats. After the sessions, intruder, and resident were kept in the same cage for 24 h separated by a perforated acrylic glass divider for continuous sensory cues. Control mice were housed pairwise in an equally divided cage. Pairings and cage were not changed throughout the CSDS period, and all mice were handled and weighed daily (Fig. 1b). In 22/26 mice from the CSDS group, the daily 5 min of physical exposure to the aggressor were recorded on video (GoPro Hero 5, GoPro) for post-hoc assessment of attack quantity and severity. Each attack of the CD1 towards the test mouse was counted and rated with a severity score from 1 (short physical contact without bite) to 3 (biting and full body contact including pinning to the ground) in score intervals of 0.5.

### Behavioral tests

#### Sucrose preference test

Mice were habituated for 2 days to the bottles and the 1% sucrose solution before the start of the CSDS period and received a similar bottle with water throughout CSDS. After the last CSDS session mice were single housed in fresh cages and received one bottle filled with water and one with 1% sucrose solution. After 24 h position of the bottles was switched and after 48 h the test ended. Consumption of water and sucrose solution was measured by comparing bottle weight at start, 24 and 48 h. Sucrose preference was calculated as the percentage of sucrose consumption and averaged between the first and second 24 h period. In accordance with previously published protocols a preference <60% was considered pathological [86].

#### Nestlet shredding test

After removal of old nest material mice received a fresh nestlet. Three hours later the quality of nestlet building was assessed by a score established by Deacon and colleagues [87] and a score <4 considered as a pathological cutoff as suggested by them.

#### Social avoidance test

Twenty-four hours after the last CSDS session the test mouse was placed in an arena (40 × 40 cm) together with an empty cylindrical wire cage and allowed to explore the arena freely while videotaping from above. After 2.5 min the mouse was removed from the arena and the empty wire cage replaced with a new one containing an unfamiliar aggressive CD1 mouse. For another 2.5 min the test mouse was again allowed to explore the arena and interact with the social partner in the wire cage. All trials were recorded on video and analyzed using Ethovision XT (Noldus). A circular area of 2 cm around the wire cage was determined as the interaction zone. Absolute time of the mouse head in the interaction zone with and without a social partner present were calculated. Cutoff for interaction time with a CD1 present was set to 50 sec based on previous publications [88, 89] and robustness compared to the social interaction rate in our setting.

### Motor learning paradigms

#### Accelerating rotarod motor learning

We adapted a method previously reported by Bilkei-Gorzo and colleagues [90]. Mice were familiarized with the rotarod device (TSE-Systems) the day before the motor learning by allowing them to walk on the rotating rod at 4 rpm until they did not fall for at least 180 sec and showed a smooth walking pattern. Motor learning consisted of 15 consecutive trials with the speed of the rotation accelerating from 4 to 20 rpm in 1 rpm/s increments and time until the animal fell was recorded. Cutoff time was 90 s per trial. Mice were allowed to rest for 60 s in-between trials. Learning curves were fitted by a sigmoid curve derived from the Hill equation. Speed of learning (LS50) equaled the number of trials needed to reach 50% of the maximum time on the rod (max. time of the learning curve).

#### Forelimb reaching motor learning and screening for paw preference

Prior to surgery mice were transferred to acrylic glass training cages (20 cm tall, 15 cm deep, and 8.5 cm wide), familiarized to the environment and offered dust-free food pellets (14 mg, TSE-Systems GmbH) after a fasting period overnight. The pellet was placed in the middle in front of the front slit so that the mouse could use either paw for reaching. This was repeated on three consecutive days with 3–5 attempts (attempts were limited to prevent early skill training). In cases where mice had used both paws, the one used more frequently was interpreted as the dominant one. For skill learning after surgery, all mice were again familiarized with the experimenter and the testing environment on five consecutive days. During the last 3 days regular food was restricted to induce ~10% loss of bodyweight to motivate reaching performance. During the following 5 consecutive days of training mice were required to reach for 48 pellets per day. The task difficulty was increased by placing a small wooden hurdle (height 1 mm) in front of the center slit, requiring the mice to reach over the hurdle. Learning success was measured as percentage of food pellets successfully eaten after the first or second correct reaching attempt. Pellets not reached at all or dropped outside of the cage as well as pellets successfully grasped but dropped inside the cage without prior eating were counted as unsuccessful trials.

### Cerebrospinal fluid sampling

We adapted this method from Pegg and colleagues [91]. Borosilicate glass capillaries were prepared with a micropipette puller (P95, Sutter Instrument) and the tip trimmed. Directly before the cardiac perfusion mice were deeply anesthetized (Ketamin 240 mg/kg and Xylazin 32 mg/kg bodyweight). Upon loss of reflexes the head was fixed in a stereotaxic frame and angled nearly 135° with the body. The shaved and disinfected skin on the back of the skull was cut medially down the neck and the dura mater of the cisterna magna exposed by blunt removal of the subcutaneous tissue and muscles. After cleaning and drying the dura mater with cotton swabs the tip of the capillary was inserted into the cisterna magna carefully avoiding rupture of the arteria dorsalis spinalis. Between 2 and 8 µl of CSF were transferred to an Eppendorf tube and snap frozen in liquid nitrogen before storage at −80 °C. In case of visible blood contamination CSF was discarded (8/46 samples, distribution 3/1/4 ctrl/resilient/susceptible).

### Perfusion and organ sampling

Mice were given a euthanizing dose of Ketamin/Xylazin i.p. as described above. The organs were transcardially perfused with 50 ml cold phosphate-buffered saline (PBS, pH 7.4). The brain was removed, the hemisphere of the cranial window transferred to ice-cold paraformaldehyde (4% in PBS, pH 7.4) overnight before dehydration in sucrose (30% in PBS, pH 7.4), freezing in isopentane on dry ice and storage at −80 °C. Adrenal glands were removed and weighed pairwise.

### Blood and fecal sampling

After induction of sufficient anesthesia using 2–3% Isoflurane a tail vein was punctured with a 27 Gauge cannula and blood transferred to a precooled EDTA microvette (Sarstedt). After centrifugation at 2000 × *g* at 4 °C for 15 min, the plasma was snap frozen in liquid nitrogen and stored at −80 °C. The mouse was allowed to recover post-anesthesia under a warming lamp. For the final sample at the end of the experiments, blood was taken from the opened heart before the transcardiac perfusion commenced.

Twenty-four hours after each mouse had been moved to a fresh cage (single-housing) after finishing the CSDS/control condition period, fecal pellets were collected from the bedding and stored at −20 °C.

### Corticosterone ELISA

Corticosterone levels in both plasma and feces were determined by an ELISA kit according to the manufacturer’s instructions (Arbor Assays, K014-H5). All samples and standards (78.128–10000 pg/ml) were tested in duplicates.

### Mass-spectrometry analysis of CSF

All chemicals from Sigma unless otherwise noted (Sigma–Aldrich Chemie GmbH, Munich, Germany).

#### Peptide preparation

Protein samples were subjected to in solution preparation of peptides with iST 96× sample preparation kit (Preomics GmbH, Martinsried, Germany) according to manufacturer’s recommendations.

#### LC-MS measurements

Peptide separation was performed on a Dionex Ultimate 3000 RSLC nano HPLC system (Dionex GmbH, Idstein, Germany). The autosampler was operated in μl-pickup mode. Peptides were dissolved in 10 µL 0.1% formic acid (FA, solvent A). One microliter was injected onto a C18 analytical column (300 mm length, 75 µm inner diameter, ReproSil-Pur 120 C18-AQ, 1.9 µm). Peptides were separated during a linear gradient from 5 to 35% solvent B (90% acetonitrile, 0.1% FA) at 300 nl/min. The nano HPLC was coupled online to an Orbitrap Fusion Lumos mass spectrometer (Thermo Fisher Scientific, Bremen, Germany). Data-dependent acquisition was performed for library generation with a gradient length of 120 min. Ions between 350 and 1500 *m/z* were scanned in the Orbitrap detector every 3 seconds with a resolution of 120,000 (AGC target 200,000). Polysiloxane (445.12002 Da) was used for internal calibration (typical mass error ≤1.5 ppm). In a top-speed method peptides were subjected to higher energy collision induced dissociation (HCD: 1.0 Da isolation, threshold intensity 25,000, stepped collision energy 25, 30, 35%) and fragments analyzed in the Orbitrap with target 80,000 and maximum inject time 50 ms. Fragmented peptide ions were excluded from repeat analysis for 20 s.

For data-independent acquisition peptide separation was performed with a gradient length of 110 min. Scan parameters were adapted from [92]. 40 windows of 15 Da plus 0.5 Da overlap were set covering *m/z* 399.5 to 1000.5. Isolated ions were fragmented with stepped HCD as above and fragments detected in the Orbitrap detector (profile mode) with a resolution of 30,000 in the range of 200–1800 *m/z*. AGC target was 500,000, maximum injection time 50 ms. Every 3 s an MS1 scan was recorded (settings as above).

#### Data analysis

Data processing was performed with Spectronaut 14.2 (Biognosys AG, Schlieren, Switzerland) either with a hybrid library approach that included DDA and DIA or without DDA data. The inclusion of DDA data yielded fewer quantified proteins. Thus, results of library-free analyses were used. Protein sequences were taken from Uniprot Mus musculus reference proteome (2020/04, 63,657 entries) along with the MaxQuant database of common contaminants (246 entries). Enzyme specificity: Trypsin/P, 2 missed cleavages, peptide length 7–52 aa. Fixed modification: carbamidomethyl on cysteine, variable modifications: acetylation on protein N-termini, oxidation of methionine. Mass tolerances were adjusted automatically. Three to six fragments were used per peptides, multi-channel interferences were excluded. Protein q-value cutoff was 0.01.

### 2-photon image analysis

The MATLAB legacy software Spine Analysis (used in publications such as [93]) available in the open-source version r3.8 of ScanImage [85] was used for quantification of dendritic spines. A spine was defined as a laterally emerging protrusion from a dendrite with a minimum length of 0.4 µm. Between 3 and 7 dendritic segments were compared in all time lapse image stacks of a region for new, lost, and stable protrusions. A custom-written python script (available from the authors upon request) was used for calculation of the reported spine dynamics.

### Immunhohistochemistry

Coronal sections (40 µm thickness) through the motor cortex were cut on a cryostat (Leica) and stored in antifreeze solution until further processing. After washing 3 × 10 min in PBS pH 7.4 blocking for 30 min in 0.1% Tween20 (Sigma) solution with 3% normal goat serum (Gibco) followed. Sections were incubated for 24 h at 4 °C with primary antibodies (anti-Iba1 1:1000, Wako 019-19741; anti-GFAP 1:1000, abcam ab4674) in blocking solution containing additional 5% bovine serum albumin (Sigma). Afterwards, sections were washed as described and incubated with their corresponding secondary antibodies (Invitrogen A-11011, A21449) at 1:1000 in 0.5% Tween20 solution for 1 h at room temperature. After washing for 3 × 20 min in PBS pH 7.4 sections were mounted on slides and protected by Fluoro-Gel mounting medium (EMS).

### Confocal image acquisition and analysis

A confocal microscope LSM 510 (Zeiss) was used for obtaining images from stained sections. Fiji (Fiji Is Just ImageJ [94]) was used for image processing and analysis.

In sections stained for GFAP z-stacks of the motor cortex (AP between 1.3 and 1.8 mm from bregma) were acquired using a ×20 objective (xy: 900 × 900 µm, 0.44 µm/px, z-step 0.95 µm). The cortex was divided into 4 quadrants resulting in 2 ROIs for layers I–III and V each, which were analyzed separately for number and morphological signs of activation of GFAP + cells following a reactivity score from 0 to 3 (Supplementary Fig. 6d) adapted and published by us [95, 96].

The Iba1 staining and GFP expression in motor cortical layers I–III were visualized by sequential imaging of z-stacks with a ×20 objective (xy: 450 × 450 µm, 0,44 µm/px, z-step 0.96 µm). Both image channels were then separated in Fiji, thresholded using the triangle algorithm and combined again in an RGB stack showing above-threshold pixels for Iba1 in red, for GFP in green and their colocalization in yellow. Within slices of each stack, segments (length ≥ 30 µm) from primary and secondary dendrites were randomly selected, set as region of interest and pixel values quantified using the histogram function. Relative colocalization of Iba1 and GFP signals was calculated by dividing the number of yellow pixels by the sum of yellow and green pixels. Morphological analysis of the Iba1+ microglia was performed using the custom-made Fiji plugin MotiQ [97] utilizing its thresholder and two-dimensional analyzer functions to obtain ramification index, total tree length, covered area and spanned area per cell.

### Statistical analysis

All behavioral scoring, motor learning, and image analysis were conducted by experimenters blinded for the treatment. The target number of mice or cells used for the individual experiments was determined based on numbers in previously published studies. No statistical methods were used to estimate sample sizes a priori. Statistical analyses were performed in Graphpad Prism Version 8.0.1. The statistical test used is indicated in the results text. The definition of n for each analysis is provided in the figure legends. Data are presented as mean ± SEM. Validity of the statistical approach was ensured by testing all data distributions for normality (D’Agostini-Pearson test). Depending on the outcome parametric or nonparametric tests were used for group comparisons. Homogeneity of variances was checked for ANOVA testing (or mixed model REML where applicable) and Greenhouse-Geisser correction was applied if appropriate. Significance was assumed at alpha = 0.05, with two-sided testing. Dunett’s post-hoc test was applied in case of multiple comparisons between control and stress phenotypes and Holm-Sidak’s test when also comparing between the phenotypes.

Five mice were excluded prior to the collection of experimental data in case of reduced quality of the cranial window (visible blurring, bleeding). Forty nine mice with successful imaging of dendritic spines on the first session (day −10) and no signs of local infection proceeded with the experiments regardless of later loss of imaging quality in the regions of interest. Only ROIs successfully imaged on day −10 and 2 were included into image analysis (Supplementary Table 1). Control mice with ≥2 behavioral results above cutoff levels (=susceptible phenotype) were excluded from further analyses (*n* = 3). Mice in the motor learning experiment were excluded before training if no reaching attempts were made during habituation (6/46, distribution 3/1/2 ctrl/resilient/susceptible) or in case a switch to the ipsilateral paw relative to the imaged hemisphere manifested within the five days of habituation (9/46, distribution 2/4/3 ctrl/resilient/susceptible).

All bioinformatic analyses were performed in the Perseus environment (version 1.6.14.0) of the MaxQuant computational platform [98]. Protein groups with fewer than two observations in each group were excluded, reducing the dataset from 2296 to 1973 protein groups. The protein group list was manually filtered for CSF sampling-introduced contaminants (collagens and keratins) and non-murine protein groups. Protein groups potentially blood-derived were not excluded as stress has been shown to alter the blood–brain barrier [99, 100]. Protein intensities were log_2_-transformed and samples were normalized by median-subtraction. For each comparison (susceptible versus control, resilient versus control, susceptible versus resilient) replicates were averaged and outliers were identified by a two-sided significant outlier detection Significance A implemented in the Perseus framework [98]. To account for multiple statistical testing the resulting *p*-values were adjusted by a false discovery rate (FDR) of 0.05 [101]. Of those protein groups with at least two-fold change were considered regulated. Protein interaction networks were built in Cytoscape version 3.8.2 [102].

## Supplementary information

complete Supplementray information

Supplementary Figure S1

Supplementary Figure S2

Supplementary Figure S3

Supplementary Figure S4

Supplementary Figure S5

Supplementary Figure S6

## Data Availability

The datasets generated and analyzed during the current study are available from the corresponding author on reasonable request.
