# Supplementary material for: Stress vulnerability shapes disruption of motor cortical neuroplasticity
Source: Transl Psychiatry. 2022 Mar 4;12:91. doi: 10.1038/s41398-022-01855-8 (PMC8897461; doi:10.1038/s41398-022-01855-8)
Supplement: Supplementary file 1 — complete Supplementray information [file 41398_2022_1855_MOESM1_ESM.docx]

# **Supplementary information**

**Supplementary Table 1**: Number of analysed regions and mice per group and imaging day (no. ROIs/no. of mice)

| **day post stress** | **-10** | **2** | **5** | **11** | **17** | ***23*** | ***29*** | ***35*** |
| --- | --- | --- | --- | --- | --- | --- | --- | --- |
| ctrl | 29/16 | 29/16 | 25/14 | 23/12 | 23/12 | *19/9* | *19/10* | *18/9* |
| resilient | 22/10 | 22/10 | 16/7 | 15/6 | 11/5 | *11/5* | *10/5* | *10/5* |
| susceptible | 16/10 | 16/10 | 13/8 | 13/8 | 13/8 | *7/6* | *5/4* | *4/3* |

**
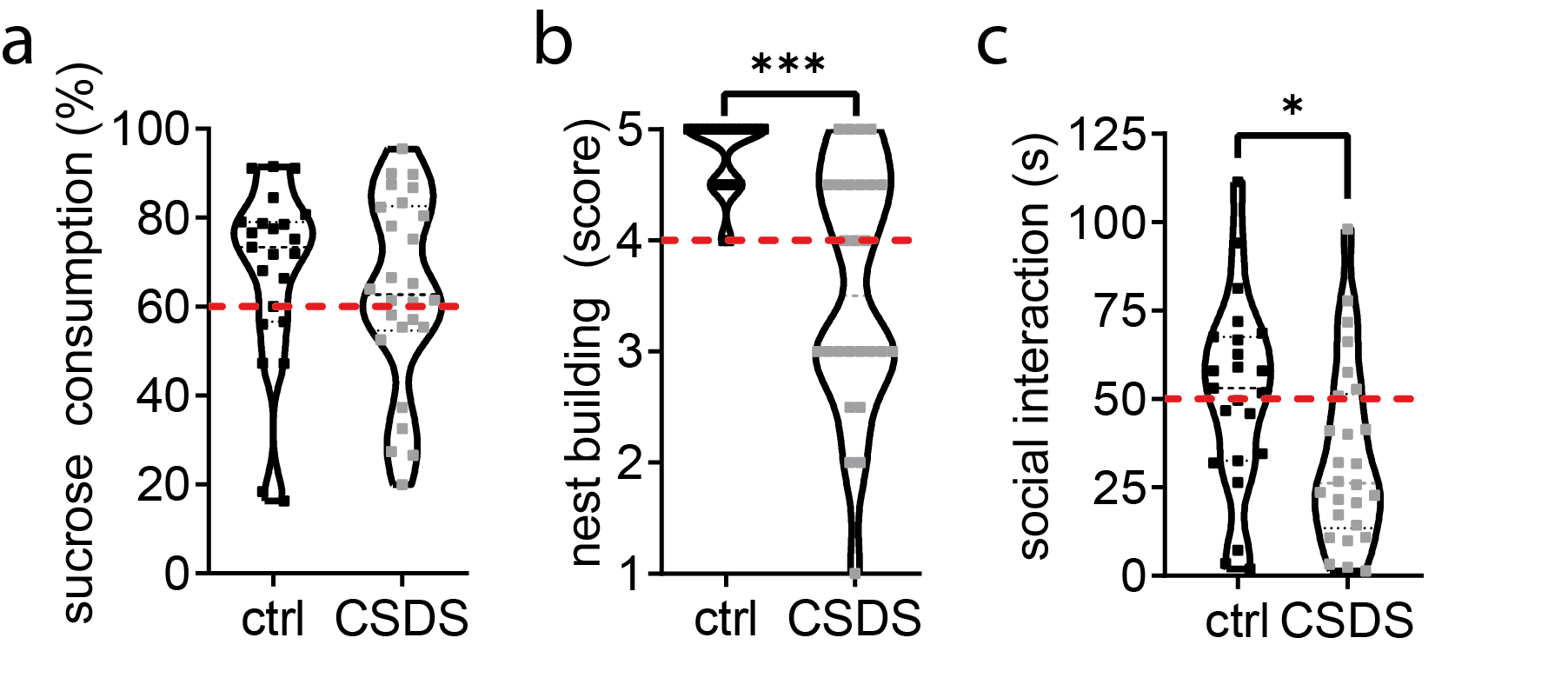
**

**Supplementary Figure 1. Distribution of individual results from the behavioral test battery.** Alternative depiction of the results from main figure 1 c (top left and right, bottom left) revealing individual values and their distribution including median (wide dashed line) and quartiles (thin dashed lines) of the datasets. Red dashed lines: cut-off as described in methods. *P < 0.05, ***P < 0.001. Results are shown as median ± quartiles.


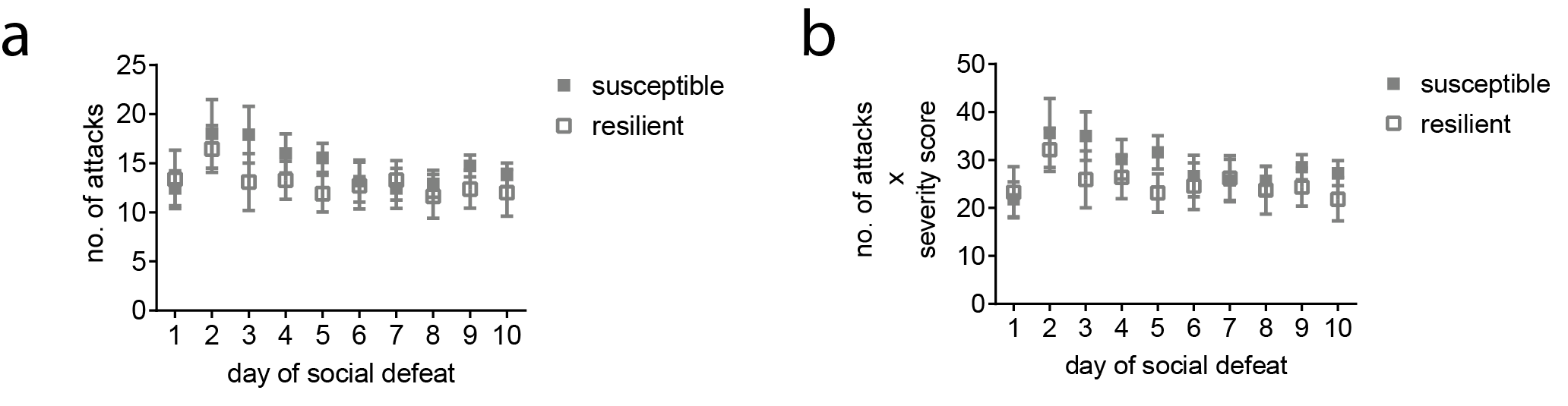


**Supplementary Figure 2.** **Quantitative and qualitative analysis of CSDS attacks.** Differences in CSDS quality as a cause for the two phenotypes could be ruled out as **(a)** daily number of attacks (time F_2.774, 55.47_ = 1.596, P = 0.203; phenotype F_1,20_ = 0.634, P = 0.435; interaction F_9,180_ = 0.571, P = 0.820; RM ANOVA) and **(b)** daily cumulative severity of attacks (time F_2.706, 54.12_ = 1.888, P = 0.148; phenotype F_1,20_ = 0.656, P = 0.428; interaction F_9,180_ = 0.514, P = 0.863; RM ANOVA) did not differ between resilient and susceptible groups. Resilient n = 11, susceptible n = 11 mice). Results are shown as mean ± SEM.

**
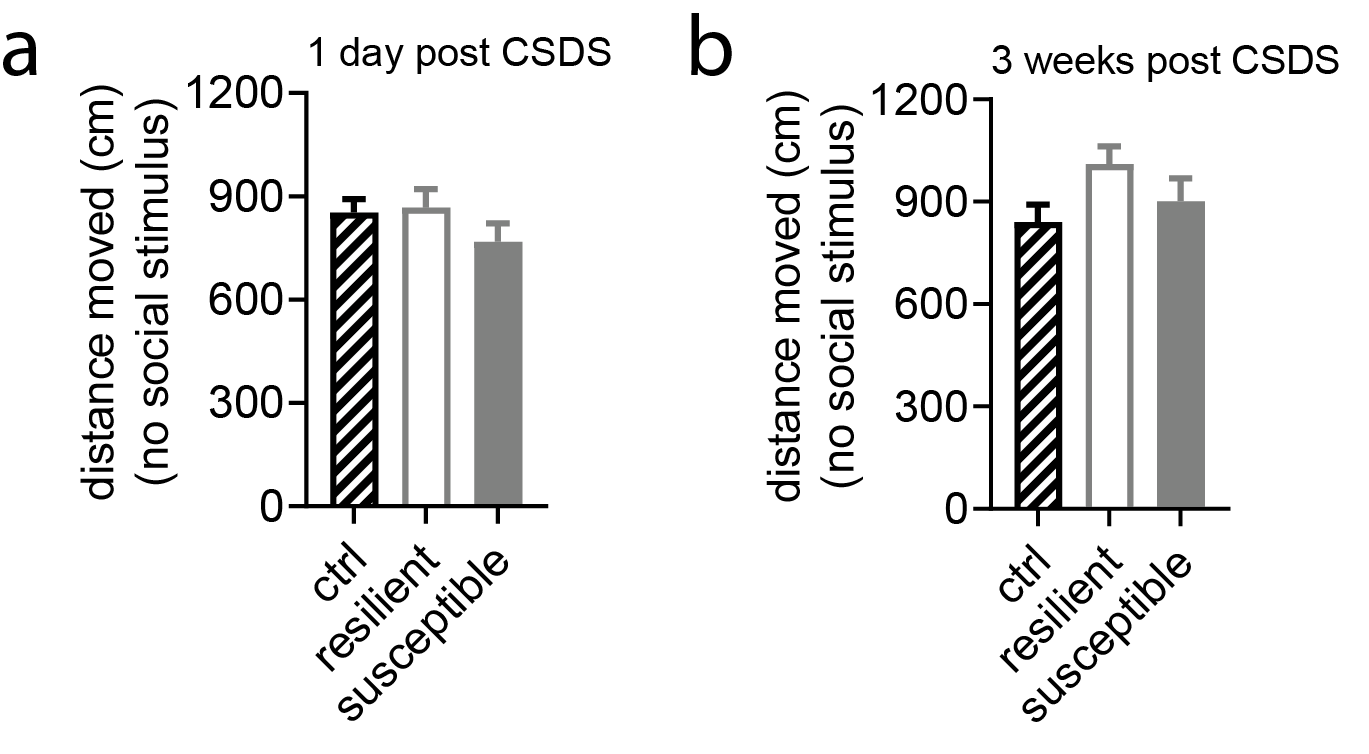
**

**Supplementary Figure 3.**  **Locomotor behavior post CSDS.** During the first trial of the SAT mice explored the arena freely without the presence of a CD1 mouse. The distance travelled served as a marker for locomotion and did not differ acutely 1 day **(a)** or chronically 3 weeks **(b)** after the stress period between the three groups (1 day post: F_2,46_ = 1.143, P = 0.328, one-way ANOVA, ctrl n = 23, resilient n = 12, susceptible n = 14 mice; 3 weeks post: F_2,43_ = 2.194, P = 0.124, one-way ANOVA, ctrl n = 20, resilient n = 12, susceptible n = 14 mice). Results are shown as mean ± SEM.


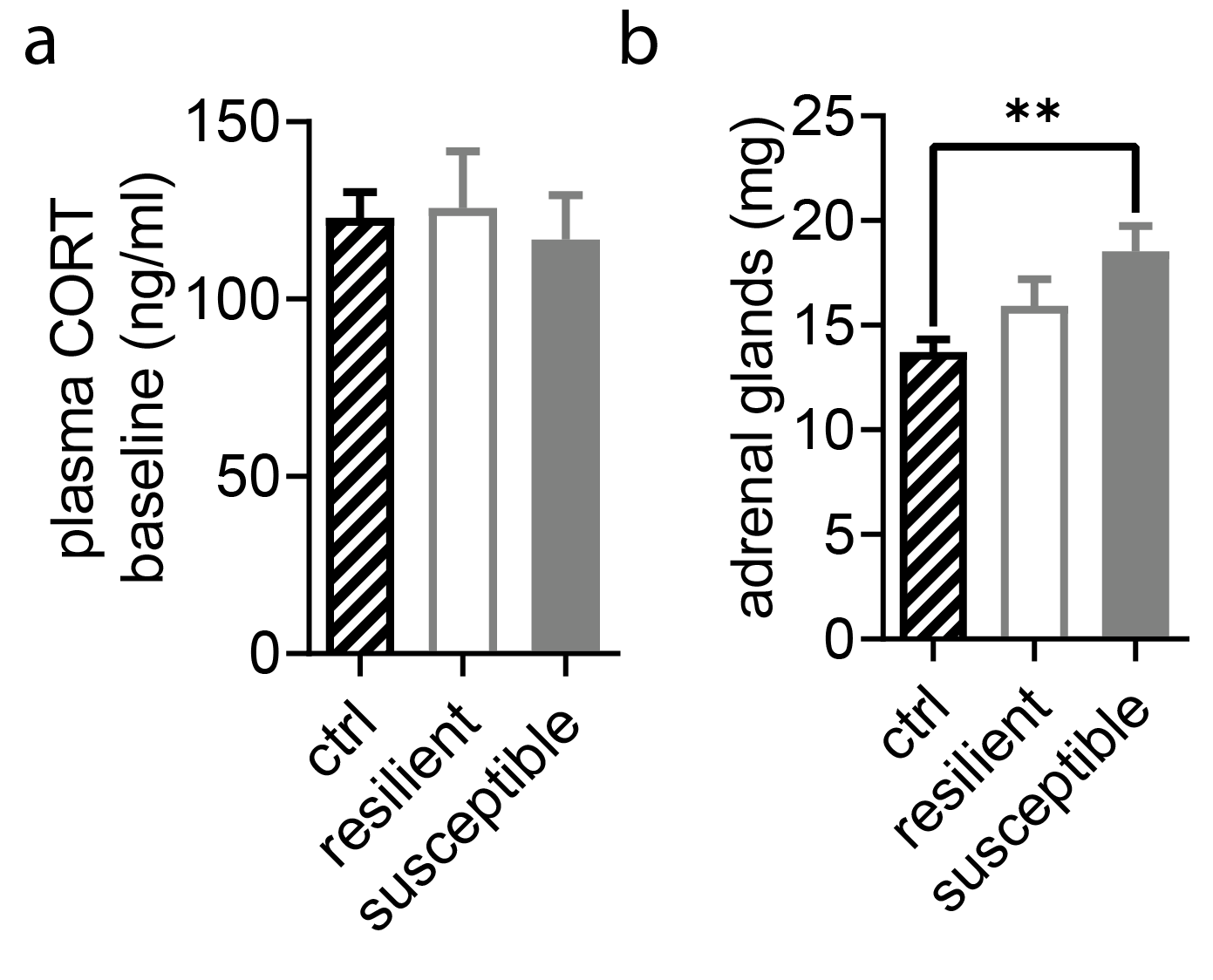


**Supplementary Figure 4.**  **Baseline plasma corticosterone and absolute weight of adrenal glands.** **(a)** Prior to the stress period group medians of plasma CORT did not differ significantly between the controls and later determined stress phenotypes (H_2_ = 0.346, P = 0.841, Kruskal-Wallis test). Ctrl n = 13, resilient n = 10, susceptible n = 9 mice. **(b)** The absolute weight of adrenal glands differed significantly between the three groups (F_2,41_ = 6.505, P = 0.004, one-way ANOVA with Holm-Sidak’s post hoc test). Ctrl n = 19, resilient n = 12, susceptible n = 14 mice). **P < 0.01. Results are shown as mean ± SEM.


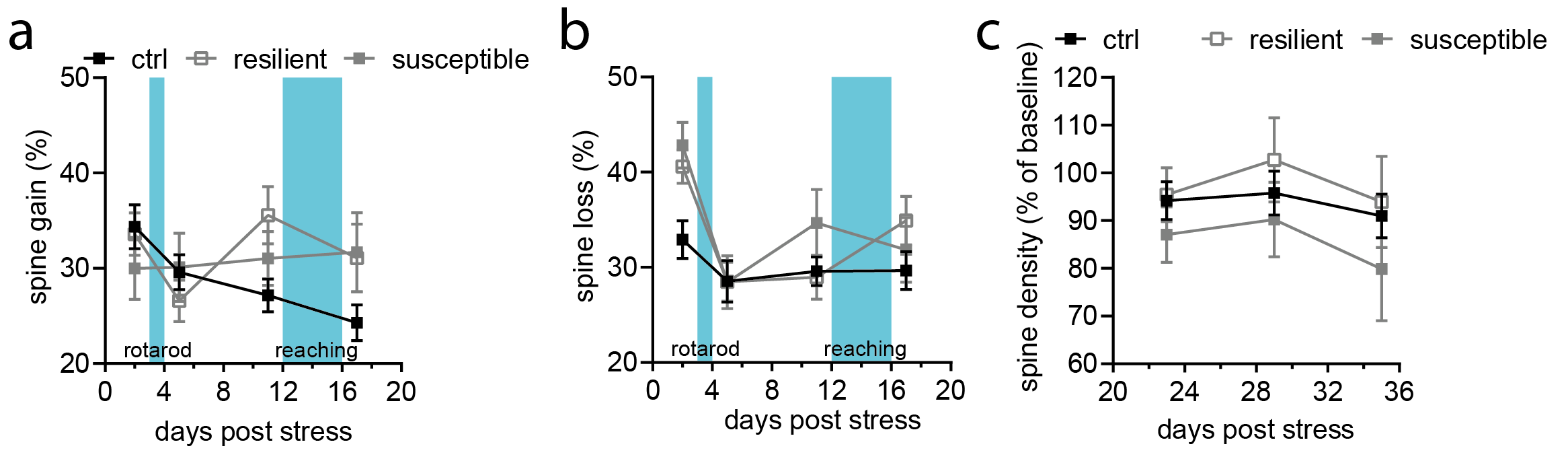


**Supplementary Figure 5.** **Spine gain, spine loss and long-term course of spine density.** **(a)** Analysis of the spine gain rate showed a significant interaction between stress and time but not for stress or time alone (time F_2.897,138.1_ = 2.667, P = 0.052; stress F_2,64_ = 0.845, P = 0.4343; interaction F_6,143_ = 2.549, P = 0.023, RM ANOVA mixed model with Dunett’s post hoc test). **(b)** Analysis of the spine loss rate showed a significant effect for time but not for stress or an interaction (time F_2.642,125.9_ = 14.15, P < 0.0001, stress F_2,64_ = 2.494, P = 0.091, interaction F_6,143_ = 1.866, P = 0.091, RM ANOVA mixed model). **(c)** The long term course of spine density between days 23-35 revealed an effect of time (F_1.894,50.20_ = 3.257, P < 0.05) but not stress (F_2,32_ = 0.6239, P ≥ 0.05) or an interaction (F_4,53_ = 0.166, P ≥ 0.05). For group sizes (number of regions/mice imaged) see STable 1. Results are shown as mean ± SEM.

**
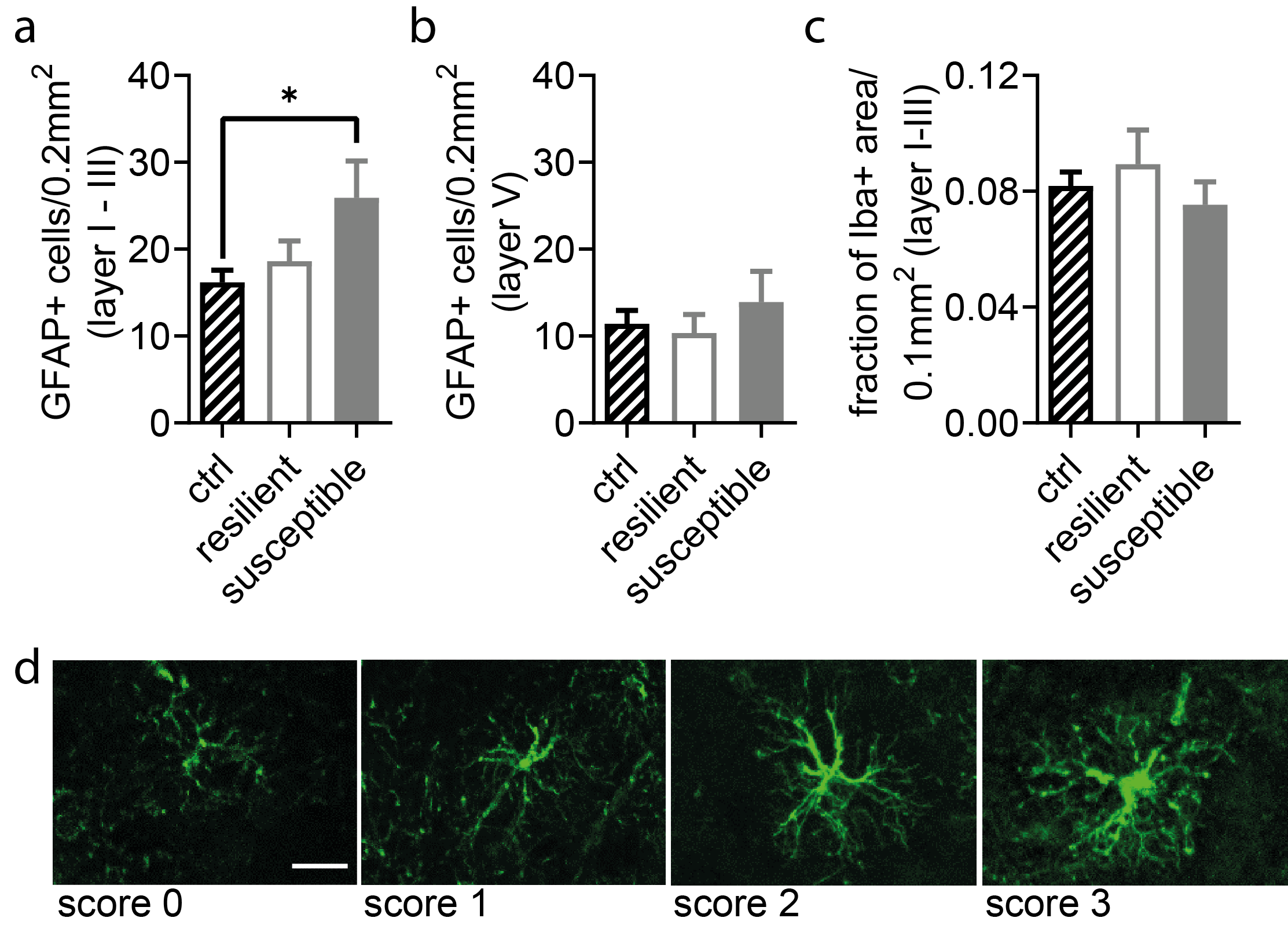
**

**Supplementary Figure 6.** **Numbers of GFAP+ cells and Iba1+ coverage in the motor cortex: (a+b)** In superficial layers I-III numbers of GFAP+ cells differed significantly between susceptible and control group (F_2,55_ = 3.908, P = 0.026, one-way ANOVA with Holm-Sidak’s post hoc test). In deeper layer V no difference was found (H_2_ = 0.873, P = 0.646). Number of analyzed ROIs/mice per group: ctrl 26/13, resilient 18/9, susceptible 14/7. **(c)** Iba1 immunostaining coverage of superficial layers I-III in M1 was not statistically different between the three groups (H_2_ = 0.4564, P = 0.796, Kruskal-Wallis test). **(d)** Example images of the reactivity score used for morphological rating of GFAP+ cells based on thickness and GFAP expression in processes and soma (0 = no reactivity, 1 = beginning thickening of processes, 2 = advanced thickening of processes, 3 = pronounced thickening of processes and soma). Scale bar: 25 µm. Mice per group: ctrl n =11, res n = 9, sus n = 6. *P < 0.05. Results are shown as mean ± SEM.
