# Supplementary figures and images for: Stress vulnerability shapes disruption of motor cortical neuroplasticity
Source: Transl Psychiatry. 2022 Mar 4;12:91. doi: 10.1038/s41398-022-01855-8 (PMC8897461; doi:10.1038/s41398-022-01855-8)

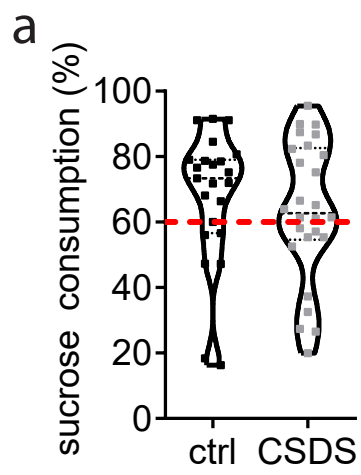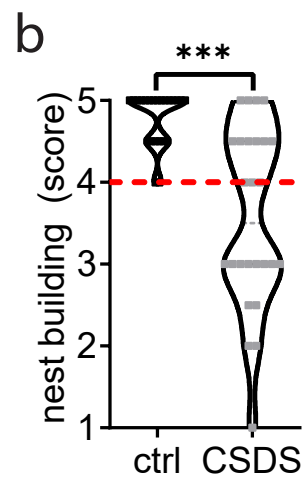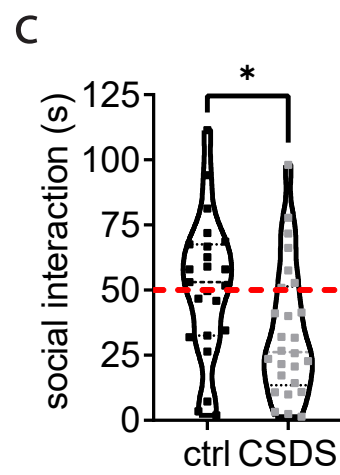

Supplement: Supplementary file 2 — Supplementary Figure S1 [file 41398_2022_1855_MOESM2_ESM.pdf]

a

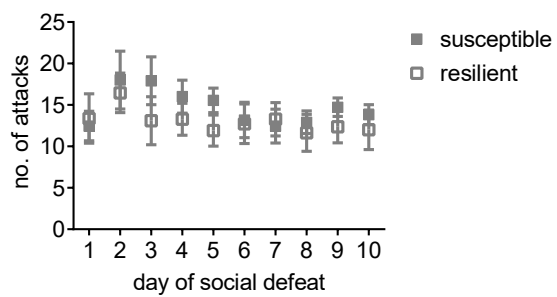

b

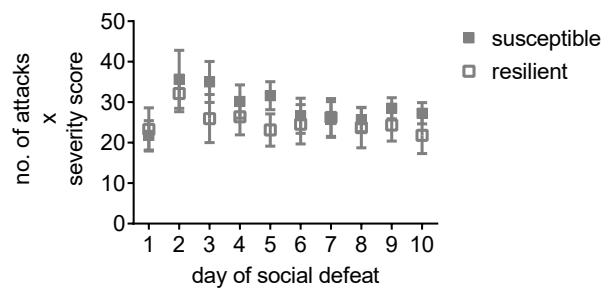

Supplement: Supplementary file 3 — Supplementary Figure S2 [file 41398_2022_1855_MOESM3_ESM.pdf]

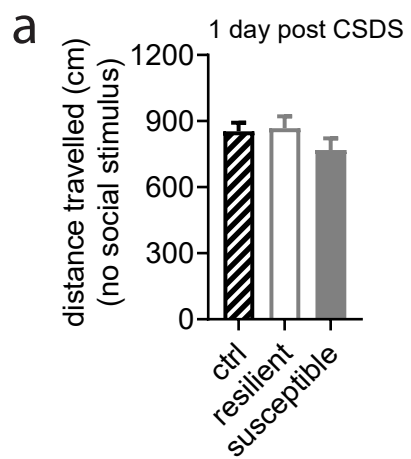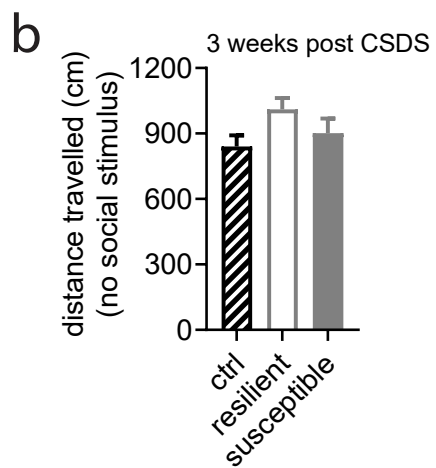

Supplement: Supplementary file 4 — Supplementary Figure S3 [file 41398_2022_1855_MOESM4_ESM.pdf]

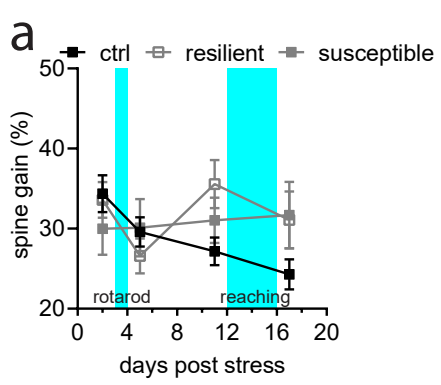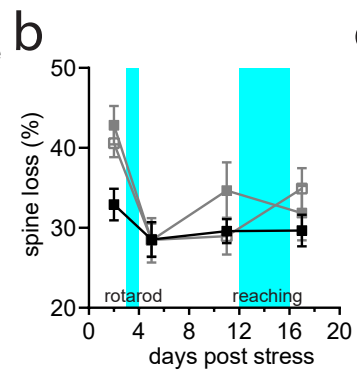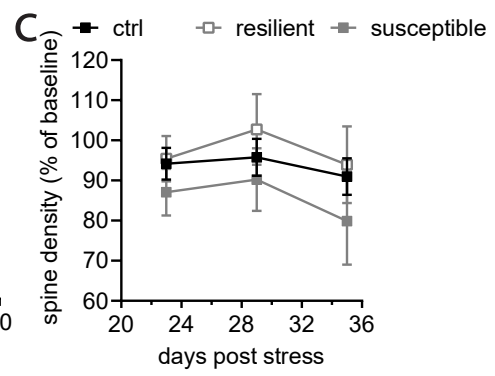

Supplement: Supplementary file 5 — Supplementary Figure S4 [file 41398_2022_1855_MOESM5_ESM.pdf]

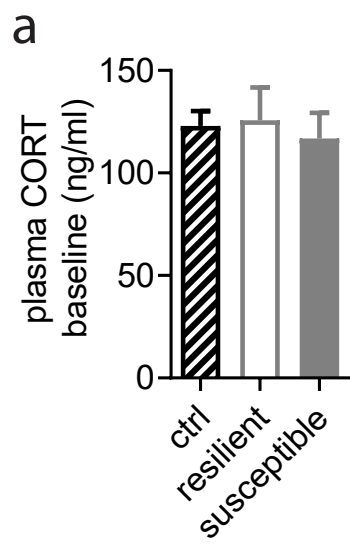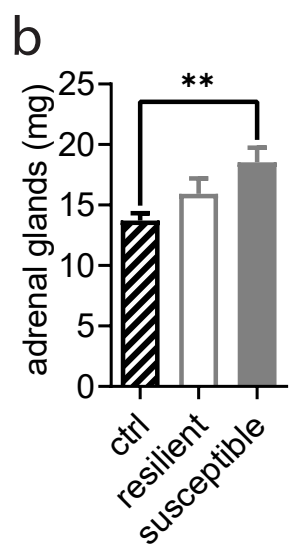

Supplement: Supplementary file 6 — Supplementary Figure S5 [file 41398_2022_1855_MOESM6_ESM.pdf]

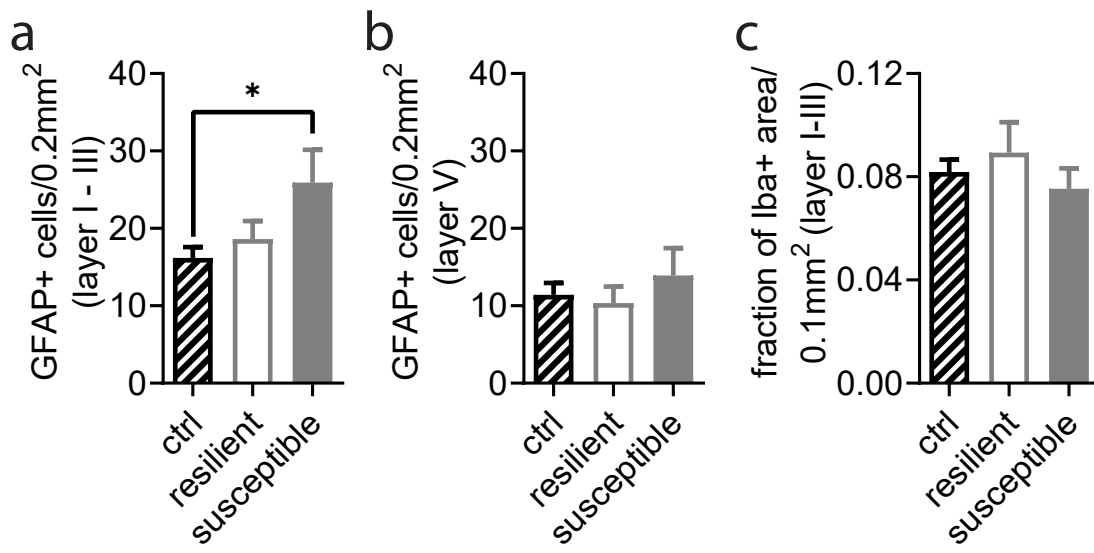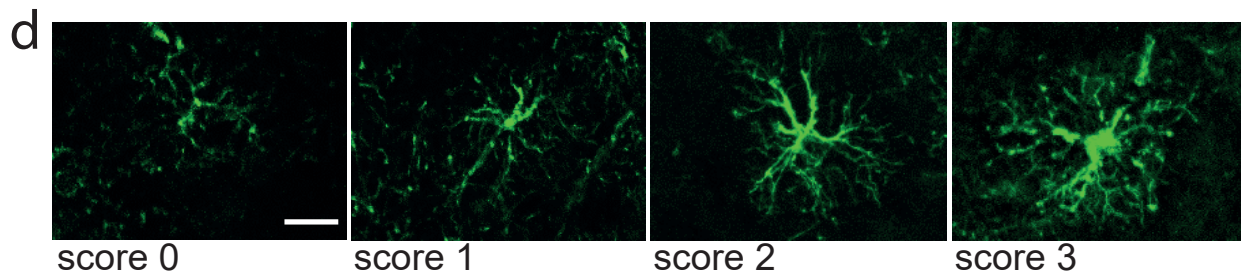

Supplement: Supplementary file 7 — Supplementary Figure S6 [file 41398_2022_1855_MOESM7_ESM.pdf]
